# Aplastic Anemia as a Roadmap for Bone Marrow Failure: An Overview and a Clinical Workflow

**DOI:** 10.3390/ijms231911765

**Published:** 2022-10-04

**Authors:** Antonio G. Solimando, Carmen Palumbo, Mary Victoria Pragnell, Max Bittrich, Antonella Argentiero, Markus Krebs

**Affiliations:** 1 Department of Biomedical Sciences and Human Oncology, Section of Internal Medicine ‘G. Baccelli’, University of Bari Aldo Moro Medical School, 70124 Bari, Italy; 2 Section of Pathology, Department of Emergency and Organ Transplantation (DETO), University of Bari Aldo Moro, 70124 Bari, Italy; 3 Department of Internal Medicine II, University Hospital Würzburg, 97080 Würzburg, Germany; 4 IRCCS Istituto Tumori “Giovanni Paolo II” of Bari, 70124 Bari, Italy; 5 Comprehensive Cancer Center Mainfranken, University Hospital Würzburg, 97080 Würzburg, Germany; 6 Department of Urology and Pediatric Urology, University Hospital Würzburg, 97080 Würzburg, Germany

**Keywords:** hematopoietic stem cells, bone marrow immune-microenvironment, bone marrow failure, cytopenia, aplastic anemia

## Abstract

In recent years, it has become increasingly apparent that bone marrow (BM) failures and myeloid malignancy predisposition syndromes are characterized by a wide phenotypic spectrum and that these diseases must be considered in the differential diagnosis of children and adults with unexplained hematopoiesis defects. Clinically, hypocellular BM failure still represents a challenge in pathobiology-guided treatment. There are three fundamental topics that emerged from our review of the existing data. An exogenous stressor, an immune defect, and a constitutional genetic defect fuel a vicious cycle of hematopoietic stem cells, immune niches, and stroma compartments. A wide phenotypic spectrum exists for inherited and acquired BM failures and predispositions to myeloid malignancies. In order to effectively manage patients, it is crucial to establish the right diagnosis. New theragnostic windows can be revealed by exploring BM failure pathomechanisms.

## 1. Introduction

Bone marrow (BM) failure can be defined as conditions in which the BM is unable to maintain normal blood count. It is not trivial to efficiently and timely distinguish the underlying cause, spanning from aplastic anemia and paroxysmal nocturnal hemoglobinuria (PNH) to hypoplastic leukemia, making the differential diagnosis of hypocellular marrow failure challenging [1,2,3]. In all these conditions, BM cellularity and architecture can be patchy. Thus, a 2 cm biopsy should always be performed, trying to avoid sampling of the subcortical region, which is often hypocellular and therefore misrepresenting in terms of BM cellularity [4,5]. Acquired aplastic anemia (AA) is a serious, although rare, condition with an incidence of 1–2 cases/million/year, (3 times higher in the Far East). AA prognosis has significantly improved in recent years thanks to advances in both diagnostic and specific supportive therapies, such as hematopoietic stem cell transplantation and immunosuppressive therapy. These have made it possible to achieve, in the most favorable cases, a 90% probability of cure. AA is defined as a condition of peripheral cytopenia with a hypocellular BM, without major signs of dysplasia, any increase in blasts, or BM fibrosis. In particular, the diagnosis of AA is based on a hematopoietic cellularity of less than 30% associated with 2 out of 3 criteria in the peripheral blood elements: (I) reticulocytes > 60,000/mmc, with automatic cell counter; (II) platelets < 20,000 mmc; (III) neutrophils between 1000–500/mmc. Neutrophil count between 500–200/mmc identifies severe AA and, <200/mmc denotes very severe AA [6].

Here, we reviewed the pathophysiology, the clinical phenotype, the diagnostic approach, and the different therapeutic options currently available for AA. Distinguishing between AA and hypocellular myelodysplastic syndrome (MDS), inherited BM failure syndrome (IBMF), and inherited myeloid malignancy predisposition (MMPS) is challenging and often not possible based on the morphological features [7,8,9,10]. Part of the challenge is that in some IBMF/MMPS cases, it is possible to observe morphologic atypia from dysplasia. An example is represented by GATA2 deficiency, an archetypic feature in the context of megakaryocytic atypia, with separated nuclear lobes or mononuclear forms, although not part of MDS. In pediatric and young adult patients with MDS, including inherited MDS, it is crucial to maintain a clinical suspicion for an underlying inherited disorder (IBMF/MMPS) since these conditions are enriched in this population [9]. Although not rigorously established, a very low CD34^+^ cell percentage determined by flow cytometry (FACS) was reported to favor AA over hypocellular MDS or IBMF/MMPS [7,8,9,10,11]. Cytogenetic studies represented a breakthrough in the diagnosis: While the majority of AA is characterized by a normal karyotype, cytogenetic abnormalities can be present. Trisomy 8 and deletion 13q can be seen in AA and in the absence of other findings and should be not considered diagnostic for MDS. Chromosome 7 abnormalities in pediatric and young adults with MDS should prompt consideration of GATA2 deficiencies and SAMD9/9L disorders. However, this discussion is beyond the scope of this overview and has been extensively examined elsewhere [12,13,14,15]; in contrast, this manuscript deals with general and specific examples of aberrant BM hematopoiesis failure, highlighting the heterogeneity and complexity underlying BM failure syndrome. From this standpoint, the approach to AA represents a roadmap for BM insufficiency. We thus reviewed the recent pathobiological advances of BM failure, dissecting the role of a systematic approach to AA to guide the clinical practice.

## 2. Approach to the Patient with Hypocellular Bone Marrow Failure

### 2.1. Focused Medical History and Physical Examination

The clinical phenotype is dominated by signs and symptoms due to cytopenia. In some cases, the medical history shows mono-linear cytopenia, seronegative hepatitis, or drug exposure. Many drugs have been associated with the origin of AA (Table 1).

After survey of the medical history has excluded the causes summarized in Table 1, including those supported by the greatest statistical-epidemiological evidence [16] and having ruled out myelophthisis as a causative role in cytopenia [17,18], the clinical methodology must be systematic. The approach to the patient with hypocellular BM failure characteristically includes complete blood cell count (CBC) as well as family history and physical exam (PE) [6]. This general workup can be useful in identifying IBMF/MMPS; however, its absence does not exclude a hereditable disorder [2]. Inherited bone marrow failure syndromes in the setting of hypocellular marrow should also be considered [6,11,19]. Nevertheless, the diagnostic process must confirm the existence of elements that define AA and exclude diseases that can enter differential diagnosis such as leukemias, hypocellular myelodysplasia (MDS), aplasia in the context of autoimmune diseases, immunodeficiencies, lymphoproliferative states, genetic-constitutional aplasia, infections [20,21,22,23], and PNH (Table 2). The initial laboratory evaluation should also consider studies recommended in all patients regardless of clinical suspicion [24].

**Figure 1:**
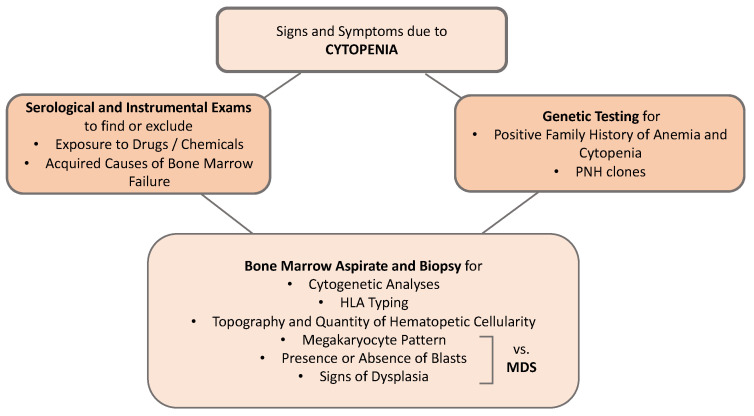
Schematic diagnostic approach to bone marrow failure. HLA: human leukocyte antigen, MDS: myelodysplastic syndromes; PNH: paroxysmal nocturnal hemoglobinuria.

A positive family history of anemia, cytopenia, or hematological or epithelial neoplasms should raise the suspicion of a genetic condition, which must also be considered in the presence of physical anomalies (small facial dysmorphism, short stature, renal and limb malformations, dystrophy of the limbs, nails, and skin). BM evaluation should include both a BM aspirate and a biopsy. The BM aspirate is used to verify the presence or absence of blasts and signs of dysplasia that when present in a marked form, are suggestive of MDS. In particular, the megakaryocyte pattern is useful in differential diagnosis, as these cells are often reduced or absent in AA, while they are small or aberrant (micromegakaryocytes) in MDS. The BM is the pivotal examination for diagnosis because in addition to verifying the presence of fibrosis and infiltrates, it defines the topography of the marrow cells and quantifies the hematopoietic cellularity, which must be <30% in children and adults, while in the elderly, who have a physiologically reduced cellularity, this cut-off is less clear. It should be remembered that in the case of tangential bone marrow biopsy, the subcortical medulla is physiologically hypocellular [6]. Although about 10% of AA patients have bone marrow cytogenetic abnormalities, the presence of a monosomy of chromosome 7 or a 5q deletion (5q-) suggests an MDS. The search for PNH clones should also be included in the diagnostic work-up of AA. Since this test can be falsely negative at diagnosis due to marked granulocytopenia, it must be repeated after any neutrophil recovery. During the diagnostic process, it is also advisable to perform HLA phenotyping, both to allow the search for a donor to start more quickly in patients who do not have an identical HLA family member and to identify subjects with HLA-DR2 and HLA-DRB1*15 who have a good chance of responding to cyclosporin A (CSA) [6,25]. The new molecular investigation methods such as next-generation sequencing (NGS) or whole-exome sequencing (WES), despite the inevitable difficulties in interpreting contradictory results, can be of help in identifying nonclassical or cryptic forms of BM failure. Therefore, they improve the diagnostic accuracy in AA [6,19,25] (Figure 1).

### 2.2. Clonal Evolution

Clonal neoplastic evolution is the most threatening event in AA, occurring in 15% of patients, and it is more frequent in elderly subjects or those with a long history of disease. It is generally heralded by a worsening of cytopenia (in responders) and by the appearance of dysplasia and/or cytogenetic abnormalities in the BM, although it should be taken into consideration that some cytogenetic abnormalities that do not include monosomy 7 can be transitory and require only follow-up [26]. The advent of new NGS techniques allowed for analyzing the genes involved in the clonal evolution of AA toward MDS. A recent study has shown that about 20% of patients with AA carry somatic mutations of genes involved in the epigenetic regulation of DNA transcription (DNMT3A and BCOR-BCORL1) or in the regulation of the immune response (ASXL1); somatic mutations are relevant in developing peripheral cytopenia and represent a molecular conundrum [27,28]. It has also been observed that BCOR-BCORL1 clones are prone to disappearing or remaining negligible and that their presence is associated with a better response to immunosuppressive therapy and a more favorable outcome. In contrast, clones with ASXL1 and DNMT3A mutations are inclined to grow over time and are associated with a lack of response to immunosuppressive therapy, and with the evolution towards MDS/Acute Myeloid Leukemia (AML). In general, patients with a shorter telomere length and longer disease duration are those at highest risk of evolution to MDS [27]. A non-neoplastic clonal evolution is marked by the emergence of PNH clones, which occurs in 40–50% of adult AA patients. Therefore, lifelong monitoring of these clones must be carried out. It should be considered that the reverse situation is also possible, namely that a patient with an active hemolytic PNH will develop AA [29].

## 3. Pathobiology

Understanding AA represented a breakthrough in understanding BM failure pathobiology. As a multifactorial disease due to an autoimmune attack on hematopoietic stem cells (HSC), AA is characterized by pathological alterations that can be identified in three discrete pathophysiological compartments that cooperate in inducing the disease: the HSC compartment, the immune niche, and the stroma milieu [19,20,30,31].

The HSC compartment is crucial because in AA patients, there is both a quantitative and a qualitative defect of HSC. The number of residual multi-potent stem cells is characteristically <1% compared to a normal subject. From a qualitative point of view, 30–50% of leukocytes in AA patients have a shortened telomere, probably due to increased cell turnover and replicative stress [32,33], which predisposes to greater fragility of the HSC. The chromosomal instability and the impaired ability to repair DNA damage represent another archetypic pathophysiological mechanism for BM failure and MDS [34,35]. From this standpoint, Fanconi anemia (FA). FA has a broad clinical spectrum, characterized by congenital anomalies, BM failure, risk of MDS/AML and increased likelihood of solid tumors [36]. Nonetheless, cryptic presentations are also possible, with the hematopoietic phenotype being impacted by genetic reversion or clonal evolution. The disease is due to chromosomal instability and an impaired ability to repair DNA damage. With high suspicion, chromosomal breakage studies and genetic testing are important [37,38].

The shortening of the telomere, however, may also be due to mutations in the genes of the telomerase-sheltering complex (i.e., DKC1, TERC, TERT) which are responsible for the repair and protection of the final portion of the chromosomes. These mutations, usually in the homozygous state, generate constitutional telomeropathies, of which classical dyskeratosis is the best-known prototype. However, mutations, especially in the TERC and TERT genes, in the heterozygous state, can be found in about 10% of AAs and could be responsible for a greater fragility of the HSC [32,39]. Both the reduced expression and the functional deficit of the transcription factor GATA2, essential for hematopoiesis can further damage the HSC fitness. These quantitative and qualitative deficits cause an increase in the adipose component of the bone marrow and jeopardize the formation of colonies of hematopoietic progenitors, thus contributing to BM failure [40,41], within a heterogenous immune niche [20,42,43,44].

As in other benign and malignant conditions, the immune niche represents the milieu where the immunological attack, a major mechanism of AA pathogenesis, takes place [5,45,46,47]. The inhibition of growth of BM progenitors in vitro mediated by the lymphocytes obtained from AA patients, or the presence in the BM from aplastic subjects of specific CD4^+^ cells and oligoclonal CD8^+^ cells corroborate these knowledges [19,45]. Confirming the pathogenic role of these clones, fluctuations correlated with the response to immunosuppressive therapy have been documented [48,49]. As crucial underlying mechanism of AA, there is an activation of self-reactive cytotoxic T lymphocytes, that release myelosuppressive cytokines including TNF-alpha and IFN-gamma, which block mitosis and increase apoptosis and hence the HSC destruction [50,51]. The formation of these self-reactive clones also seems to be favored by the lack of regulatory T lymphocytes (Tregs) in AA patients, being also qualitatively altered [52,53]. Furthermore, in AA subjects, Kordasti et al. used mass flow cytometry (Cytof) and identified two subpopulations of Tregs (A and B) with a different phenotype, gene expression and function [54]. Type B predominates in patients who respond to immunosuppressive therapy, have a memory/activated phenotype, and express the interleukin-2 (IL-2/STAT5) pathway, and are therefore sensitive to IL-2. These data are critical because they allow for the identification of predictive markers of response to immunosuppressive therapy and suggest new therapeutic approaches such as the use of low dose IL-2 [54]. A potential pathogenic role is also exerted by the overexpression of ligands such as NKG2D in HSC. These molecules fuel a vicious cycle, inducing BM homing of T, NK, and NKT lymphocytes. These cells, upon activation, can in turn damage the HSC compartment [55].

The presence of polymorphisms in the promoter regions of the myelosuppressive cytokine genes in patients with AA also suggests a genetic influence in the immune response that, in subjects who are carriers, can hijack the inflammatory response throughout an uncontrolled cytokine-mediated BM inhibition [56]. In contrast, the genetic landscape can shape the immune response in a dynamic and context-dependent fashion: specific HLA alleles such as DRB1 (1501, 0405), B14, B (4801) seem to support the development of AA, while others (HLA-DRB*03:01, HLADRB1*11:01, HLA-DRB1*03, HLA-B*51:01) may have a protective role [57]. The complexity of the genomic makeup is also confirmed by the acquired loss of heterozygosity of the short arm of chromosome 6 (6pLOH), uniparental disomy of chromosome 6 and by the mutations of HLA genes (i.e., HLA B40.02). The lack of expression of HLA molecules on HSCs in turn is the driver of a deficiency in the immune recognition process [58,59]: The mutant HSCs survive the immune attack, thus generating an oligoclonal hematopoiesis that could drive the residual BM proliferative activity [60,61,62].

**Figure 2:**
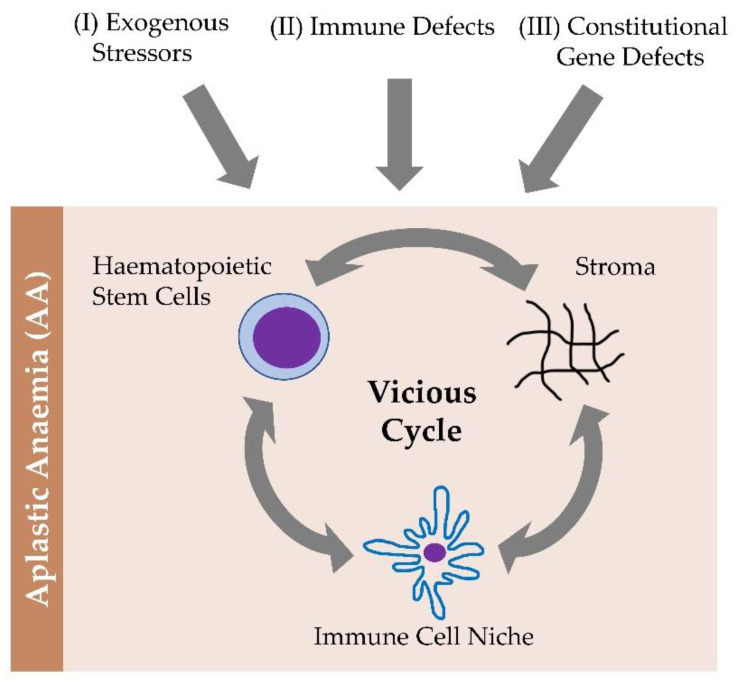
The pathobiological landscape and a schematic overview of aplastic anemia.

From the stroma perspective, AA individuals are characterized by a failure in a maturation arrest, halting T lymphocytes and the production of myelosuppressive cytokines such as IFN-gamma. This aberrant phenotype cannot be corrected by immunosuppressive therapy, but can be reversed by BM transplantation [63]. The current multifactorial pathogenic model of AA, while presenting aspects that are not completely understood, contemplates an initial event that can be a viral infection or a genetic mutation in the compartment of the HSCs that determines either the generation of neo-antigens or an incorrect presentation of an antigen by the major histocompatibility complex. This leads to the formation of self-reactive T cells that expand and release myelosuppressive cytokines including TNF-alpha and IFN-gamma. By causing excess apoptosis of the HSC, these trigger a depopulation of the hematopoietic component of the BM [20]. Indeed, IFN-gamma has a strong impact on MAPK signaling, thus influencing several biological functions in both malignant and non-malignant diseases, including AA [64,65,66]. The pathogenic role of self-reactive T cell clones specific to the glycosylphosphatidylinositol (GPI) complex expressed on the HSC has recently been documented in subjects affected by AA. GPI absence determines complement-mediated lysis of the red blood cells, that generates the intravascular hemolysis characteristic of active PNH. This allows the identification of these molecules as one of the targets of the autoimmune attack on the BM, thus explaining, also the emergence and coexistence of PNH clones (which are GPI negative) with AA in addition to the onset of BM failure, frequently observed in the course of the disease [29,67]. Indeed, the differentiation between AA, PNH and, sometimes, hypoplastic leukemia, are not always trivial (Figure 2).

Trisomy 8 and deletion 13q can be seen in AA, chromosome 7 abnormalities in pediatric and young adults with MDS should prompt consideration of GATA2 deficiency and SAMD9/9L disorders [12,13,14,15].

### Defects in the Innate Immune Stress Response

Initial defects in the innate immune stress response can be activated by a plethora of intrinsic and extrinsic pathways [19,68]. Those factors promote the activation of autoreactive T-cells. This seems to be a common sequence of events to many pathological states, both proliferative and degenerative [69]. Despite different antigen have been postulated to trigger the immune attack against the HSC in AA, the insights provided by the knowledge of innate immune response have largely boosted the landscape contributing to the immune pathogenesis of BM failure [20,70,71,72]. As a result of chemical agents, drugs, and viruses, acquired BMF syndromes result from intrinsic direct or indirect damage to the HSC pool. Viruses or drug metabolites can also cause indirect injury to HSCs, but this depends primarily on immune effector mechanisms. There are qualitative as well as quantitative impairments of the self-renewal ability of HSCs with constitutional and acquired BMF syndromes, irrespective of the initial injury to the BM. As stem cells maintain themselves over time, they are able to differentiate and mature into mature circulating cells. This is called self-renewal, which enables stem cells to maintain hematopoiesis for a long time. In addition to several membrane-bound and soluble cytokines within and outside the hematopoietic niches, a complex network between hematopoietic and stromal cells controls hematopoiesis. As a result of acquired BMF syndromes including acquired AA, hypoplastic myelodysplastic syndromes (hMDS), chronic T-cell and natural killer-granular lymphocyte diseases, it has been widely reported that cell and cytokine interactions and cooperativity are disturbed [57,72].

Although most studies have focused on T and B cells, dysfunctional innate immunity may also contribute to AA pathogenesis. NK cells play a conflicting role in AA, according to disputing evidence [73]. It has been shown that in AA, NK numbers and cytolytic activity are severely reduced, and improvement in NK function is correlated with improvement in haemopoietic function after IST [74]. Patients with AA may have a deficiency in NK cytolytic activity due to intrinsic mutations in the perforin gene or because autologous granulocytes suppress NK function [74,75]. However, the frequency of NK cells in pediatric AA patients did not correlate with the severity of the disease or response, raising the question of whether bone marrow failure causes NK cell deficiency. The UL16-binding protein (ULBP) ULBP1, ULBP2, and ULBP3 ligands of the natural killer group 2 member D (NKG2D) are abnormally expressed on granulocytes and bone marrow cells, and the major histocompatibility complex class I chain related molecules A (MICA) is abnormally expressed. Bone marrow failure and a favorable response to IST in patients with AA are positively associated with NKG2D ligand expression on granulocytes. A research study revealed that in vitro studies involve autologous lymphocytes bearing NKG2D, including NK, NK T, CD8^+^, CD4^+^ and a small subset of CD4^+^ T cells, which damage AKHA hematopoietic progenitor cells with abnormal NKG2D ligand expression, consistent with findings in PNH [76,77,78]. In conclusion, these findings suggest that NKG2D-mediated immunity plays a role at least partially in the pathogenesis of AA since it drives NK, NK T and T cell activation. Immune-mediated marrow damage may be detected early by detecting NKG2D ligands on hematopoietic cells, and immunosuppressive therapy response can also be predicted [77,78].

## 4. Treatment

The treatment of AA must be carried out in specialized centers with proven expertise in the management of patients with BM failure. During the diagnostic process, it is necessary to stabilize the patient from the standpoint of hemorrhagic and infectious control. It is not recommended in this preliminary phase to start steroid therapy, which is ineffective on hematopoiesis at the same time that it can increase the risk of infection. Instead, it is necessary to start supportive therapy, despite avoiding side effects [79,80,81]. The specific treatment aims to restore hematopoiesis either through hematopoietic stem cell transplantation (HSCT) or through combined immunosuppressive therapy (IST).

### 4.1. Supportive Therapies

Anemia must be treated with concentrated red blood cell transfusions with the aim of correcting the symptoms due to anemia rather than reaching a specific hemoglobin value. Martial overload should be monitored, and chelation initiated if the iron load on magnetic resonance imaging (MRI) T2 is >200 mL/kg or liver iron content is >7 mg/g of dry weight. If it is not possible to evaluate hepatic iron content with MRI T2, then serum ferritin, although less specific, is an acceptable marker of iron overload indicated by stable values > 1000 ng/mL [26]. Thrombocytopenia should be treated with platelet concentrates only when the count is <10,000/mmc or in the case of active bleeding. Platelet transfusion below the value of 30,000/mmc during therapy with anti-lymphocyte globulin (ATG) is debated [82]. All transfused blood products must be leukodepleted and irradiated to reduce the risk of transfusion reactions and sensitization. In addition, pneumocystis prophylaxis is recommended in lymphopenic patients. Antifungal prophylaxis may be considered if the neutrophil count is persistently <200/mmc. Antibiotic prophylaxis should be considered in patients with neutrophils <200/mmc between day +30 and +90 after immunosuppressive therapy [83].

### 4.2. Specific Therapies

Patients with severe AA and patients with non-severe but transfusion-dependent AA should be treated as quickly as possible with specific therapies since in such circumstances, spontaneous remission is extremely rare, and an interval of more than 2–3 months between the diagnosis and treatment onset is associated with a worse prognosis [84,85,86]. An identical HLA family donor HSCT is the first-choice option in pediatric patients and young adults. The rationale for IST is to counteract the autoimmune attack on BM hematopoietic cells using anti-lymphocytic drugs (anti-lymphocyte serum, cyclosporin). This therapy offers long-term survival probabilities similar to those of a compatible family member transplant [87,88] but a worse survival rate, especially in pediatric patients [89,90,91].

#### 4.2.1. Hematopoietic Stem Cell Transplantation: Identical HLA Family Donor Transplant

The identical HLA family donor HSCT induces excellent results, with an overall survival probability between 76% and 90%; the probabilities of graft failure, acute and chronic GvHD, or transplant-related mortality are about 10%, and the risk of secondary cancer is less than 1%. In pediatric patients, event-free survival (EFS) as a qualitative indicator is about 85% and significantly higher than that obtainable with IST (33%) [89]. On the basis of these data, HSCT from identical HLA family members should be considered the first-line therapy in pediatric patients and in young adults aged up to 40 years [85,87,89,92]. HSCT behaves in much the same way in patients up to 50–60 years in cases of severe aplasia if the subject is in good general condition and without comorbidities [87,93]. The most used conditioning regimen under the age of 30 includes cyclophosphamide 6 g/m^2^ (total dose) + ATG. In this context, rabbit ATG resulted in a lower incidence of acute and chronic GvHD [94]. For the ages between 30 and 50–60 years, the reduced intensity FCC regimen is suggested (Fludarabine 20 mg/m^2^, Cyclophosphamide 1200 mg/m^2^, Campath 60 mg total dose) [93].

#### 4.2.2. Hematopoietic Stem Cell Transplantation: HSCT from a Non-Familial Donor Identical HLA (MUD)

The use of modern reduced-intensity conditioning regimes facilitated donor selection due to the improvement of HLA typing techniques and the advances in supportive therapies. These have led to a significant improvement in results. Therefore, this type of transplant is used in young individuals as first-line therapy in the absence of an identical family HLA donor. A retrospective controlled English study conducted in collaboration with the Severe Aplastic Anemia Working Party (SAAWP) of the European Society for Blood and Marrow Transplantation (EBMT) on pediatric patients (median age 8.6 years) undergoing HSCT from MUD in first line [90], demonstrated survival and EFS probabilities of 96% and 92%, respectively. These data are comparable to those obtained with HSCT from an identical HLA family member in the first line (91% and 87%, respectively) and to the probability of survival determined by IST (94%) in the first line. Conversely, they are significantly better than the EFS (40%) obtained with IST as front-line therapy. Subsequent retrospective studies on patients aged < 20 years [95,96] confirmed these data.

Notably, both survival and EFS of first-line HSCT MUD are statistically superior to those found in subjects treated with HSCT MUD after IST failure (74% for both), which suggests that it is important to avoid waiting for the HSCT MUD failure. Thus, it is reasonable to act proactively avoiding transplant failure. IST in the front line to attempt a recovery with HSCT MUD which, although satisfactory, is inferior to the same option practiced as a first line of therapy [90].

The conditioning regimen used in the English study included Fludarabine (total dose 120 mg/m^2^), Cyclophosphamide (total dose 1200 mg/m^2^) and Campath (0.9–1 mg/kg).

Based on these data, it appears legitimate to consider, in subjects < 20 years without a family donor, MUD transplantation as a first-line option. MUD should be performed with an identical donor for 10/10 or 8/8 Ag HLA within 2–3 months of diagnosis to avoid the risk of serious infections linked to the persistence of neutropenia. This is also instrumental for ensuring survival and minimizing the risk of clonal evolution. For those who do not have these conditions, IST is still a good option, although it does require the lifelong monitoring of the risk of neoplastic evolution [87].

Even in adults, the results of HSCT MUD have greatly improved in recent years. For individuals between 10–30 years without comorbidities, the probability of survival approaches 80% [87,93]. However, most of these data were obtained in patients who underwent transplantation after IST failure and therefore cannot be considered robust enough to generate the indication for HSCT MUD in the first line even in adults with an identical HLA family donor. In adults, the indication for HSCT MUD depends on various factors such as age of the recipient; indeed, the probability of survival was 49% in patients older than 40 years, and it progressively shortened with age [87]. The presence of comorbidities correlates with a lower probability of survival, estimated in about 40%, in subjects with a higher Charlson Comorbidity Index [93]. The age of the donor is also associated with improved survival, indicated by a lower risk of infection and a lower risk of GvHD with donors aged < 40 years [97]. Moreover, the telomere length of the donor is also positively correlated with prolonged survival [98]. In much the same way is the clinical scenario regarding the degree of HLA compatibility and the CMV status and the cellular source used. In these cases, there is a significantly better survival rate of about 90% with the use of BM cells compared with peripheral blood cells, with estimated survival <75% [99]. Basically, the indication for HSCT MUD must be evaluated case by case and based on the expertise of the center. To support this decision, risk assessment systems are available that take into account the aforementioned factors in various combinations [100,101].

In summary, in adults, HSCT MUD, even if the results are continuously improving, cannot be considered a first-line option. Instead, it represents a good option if there is a young donor identical for 10/10 or 8/8 Ag HLA, in young adults who have failed IST, who are in good clinical condition and without comorbidities. The most used conditioning regimen in such circumstances involve fludarabine (total dose 120 mg/m^2^), cyclophosphamide (total dose 480 mg/kg), ATG, and TBI 2Gy if age > 14 years or even under 14 years if the patient has received multiple transfusions.

#### 4.2.3. HSCT from an Alternative Donor

In recent years, transplants from haploidentical family donors have progressively increased, and this concept was also used in AA. Basically, two platforms have been applied. The first employs peripheral HSC manipulated to reduce the risk of GvHD by reducing the alloreactive cells towards the recipient (Tαβ^+^ cell depletion), to halt the risk of EBV lymphoproliferative syndrome (with the reduction of CD19^+^ cells), but to preserve the anti-infective activity (maintaining the NK cells, the T γδ^+^ and the monocytes). Hence, all the efforts are finalized to avoid an engraftment failure, as the cellular product is highly enriched with HSC. The second platform is based on post-transplant cyclophosphamide with the aim of targeting the alloreactive activated T cells of both the recipient and the donor. The main advantage of this type of transplant is the rapid availability of the donor. The results obtained so far are encouraging [102], nevertheless have been reproduced only in a limited number of centers so that it cannot be considered a standard of care [87].

### 4.3. Immunosuppressive Therapy (IST)

The immunosuppressive therapy (IST) of choice consists of ATG combined with CSA which has proven to be more effective than ATG alone [84].

The addition of a third immunosuppressive agent to this combination, such as mycophenolate or sirolimus, with different mechanisms of action and theoretically able to enhance the immunosuppressive effect, showed no advantage in either response to treatment (62% at 6 months) or prevention of relapses [103]. Therefore, the association of ATG and CSA, combined with the steroid, which is not curative but has the sole objective of preventing serum sickness, currently remains the first-choice immunosuppressive therapeutic option.

Regarding the source of ATG, the most important prospective randomized study [104] showed that equine origin seems more effective than that derived from rabbits (survival at 3 years of 96% over 66%), which also implies a high incidence of early deaths. This “outcome” was confirmed by an EBMT study [105] that recorded an OS of 91% for patients with severe AA treated with horse serum compared with 73% of those treated with rabbit serum, showing a higher incidence of late fatal infections in the latter group than in the American study. Two recent independent real-world studies [106,107] have substantially confirmed this hierarchy. Therefore, based on these data, horse ATG appears to be the most effective source; moreover, after having been unavailable for a long time in Europe, it is now again usable in many continental countries. Horse ATG should be administered at a dosage of 40 mg/kg/day for 4 days, associated with CSA, which should be started on day +1, at a dose of 5 mg/kg/day split into two daily administrations. The full dosage of CSA adjusted based on serum levels, which must be between 100 and 250 ng/mL, should be maintained for 12 months. After this period, if complete response is achieved and maintained, CSA should be slowly reduced (5–10% dose reduction every month) over the next 12 months for a total therapy duration of 2 years. In fact, it has been shown that a slow and gradual reduction of CSA is associated with a reduction in the risk of relapse compared to a more rapid tapering [108]. If during the “tapering” of the CSA a drop in values is observed, the first measure is to bring the CSA back to the full dose.

The use of G-CSF, after treatment with ATG and CSA, reduced the infectious risk in the first periods after IST while awaiting the hematological response. It has also been observed that G-CSF reduces the rate of infections and days of hospitalization, without affecting survival, EFS and response to therapy [109]. The same study demonstrated that G-CSF can predict response since patients who received G-CSF and achieved >500 neutrophils/mmc on day 30 were more likely to respond to IST after 4 months [109].

The role of G-CSF favoring clonal evolution towards MDS/AML is not unequivocally demonstrated. A retrospective study of EBMT SAAWP of 840 patients treated with ATG and CSA, of whom over 43% also received G-CSF, showed that the use of this drug was associated with an increased risk of MDS/AML (10.9%) in those who were exposed to G-CSF compared with those who were not (5.8%) [110]. However, not even meta-analyses [111], i.e., the highest level of medical scientific evidence, have as yet confirmed an increased risk of clonal evolution associated with the use of G-CSF. Given all these data, it is reasonable to minimize the use of G-CSF by confining it continuously to the first 30 days of treatment and then “on demand”, in the case of infections in neutropenic patients, in the following days. Recently, the National Institutes of Health (NIH) group tested the association of classical IST therapy (CSA and equine ATG) with the drug Eltrombopag, a thrombopoietin receptor agonist (TPO) capable of stimulating the residual hematopoietic component [103]. The addition of this substance improved the response obtained with classical IST alone, reaching overall response values (partial and complete) at 6 months of 86%. In the group of patients in which Eltrombopag was administered simultaneously with the first day of ATG, the probability of overall response was 94%, with 58% complete responses at 6 months. The risk of clonal evolution was comparable to that of previous immunosuppressive protocols. The results of this prospective but not randomized study need to be confirmed by the ongoing randomized trial in the SAAWP of EBMT, where the arm with classical IST (Horse ATG and CSA) is compared to the arm with the same drugs but with the addition of Eltrombopag (RACE trial) [112].

Treatment with IST is characterized by a risk of hematological neoplastic clonal evolution (MDS/AML), which ranges in the various studies from 8 to 21% at 10–15 years and which is greater in unresponsive subjects than in those who suffer recurrence after having responded [50,108,113,114]. A recent study on a population of adult patients only has also documented an increased risk (14% at 20 years and 24% at 30 years) of non-Hodgkin’s lymphomas and non-hematological neoplasms, such as breast, urothelial and colon cancers. Long-term risk of cancer development in adult patients with idiopathic AA after treatment with anti-thymocyte globulin [115].

Considering the above data, IST remains the first-line therapy in all subjects > 60 years of age, in pediatric patients and in young adults who have no identical HLA donor, either familial or not, or who are not eligible due to comorbidities for HSCT MUD. For the above reasons, it is necessary to monitor the “life-time” risk of neoplastic evolution, especially in those who have not responded to IST or have relapsed after the initial response.

### 4.4. Forms Refractory to First- and Second-Line Therapies

In the event of IST failure and in patients not eligible for HSCT from an identical HLA family member or MUD, only less effective alternatives are available, beyond haploidentical transplantation [103]. High-dose cyclophosphamide represents an option, despite previous evidence that high response rates were associated with a higher incidence of serious infectious events.

Although their efficacy has not been confirmed in randomized studies, based on some experiences, their possible potential can be hypothesized in patients refractory to IST. The treatment must be continued for at least 3 months before being declared ineffective.

Alemtuzumab (anti-CD52 monoclonal antibody) has been associated with low-dose CSA. This combination has shown promising results in a study conducted by the SAAWP EBMT in adult patients, with a response rate of 58% without severe toxicity. This latter scenario was also confirmed in a NIH study that instead showed different response rates based on the phase of the disease (53% in relapses and 37% in refractory forms).

Eltrombopag was evaluated in a phase II study, and it achieved activity in 44% of 25 adult patients refractory to IST, in some cases achieving trilinear effectiveness. In a follow-up update of subjects treated with Eltrombopag, 8/43 (18.6%) developed clonal cytogenetic abnormalities after the drug administration. A French study [116] showed that Eltrombopag monotherapy in patients who were refractory or relapsed after IST determined a unilinear response in 74% and a multilinear response in 34% of cases.

### 4.5. Moderate Aplasia

Patients with transfusion-independent moderate AA can achieve spontaneous remission without specific treatment [117]. Transfusion-dependent patients, on the other hand, can progress to the severe form or remain stable for months or years.

Given the potential toxicity of specific therapy and the lack of a high level of evidence of the benefits of early initiation, a period of observation and supportive therapy, followed by specific treatment only in patients with transfusion-independent AA, appears reasonable.

### 4.6. Treatment Selection and Follow-up Summary

**Figure 3:**
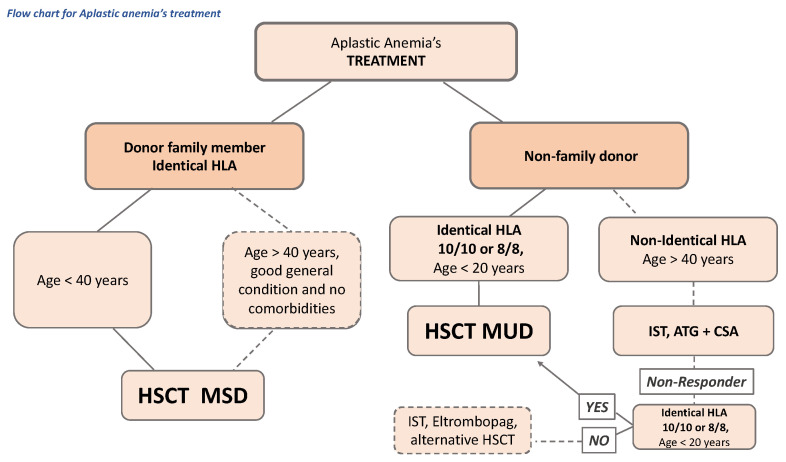
Schematic treatment selection and follow-up in AA. CSA: cyclosporine. HSCT: hematopoietic stem cell transplant. HLA: human leukocyte antigen. IST: immunosuppressive therapy. MSD: HLA-matched sibling donors.

There is a wide phenotypic spectrum for inherited BM failure, AA, and myeloid malignancy predisposition syndromes. They should be considered in the differential diagnosis of both children and adults with unexplained defects in hematopoiesis. A detailed family history, physical exam and selected testing are essential to establish the correct treatment allocation plan for patients with AA (Figure 3), as well as the screening for malignancy and family planning.

## 5. Aplastic Anemia and Paroxysmal Nocturnal Hemoglobinuria: Outlook

As previously indicated, the appearance of a PNH clone occurs in 40–50% of patients with AA. The presence of PNH clone does not change the AA treatment algorithm unless the hemolytic/thrombotic component becomes clinically evident (increased LDH, hemoglobinuria, bilirubin, further anemia or increased transfusion requirements, decreased ferritin, thrombotic events). In this case, it is necessary to introduce therapy with Eculizumab, a monoclonal antibody anti C5 complement fraction, which has proven to be effective in PNH subjects on intravascular hemolysis, with anemia, or transfusion-dependent [118] and at thromboembolic risk [119], accounting for about half of the subjects, resulting in an overall survival probability of >90% [120]. Therefore, monoclonal antibodies offer a novel promising theragnostic window. The clinically evident association of PNH with AA can be treated with the eculizumab and IST combination, used either concomitantly or in succession [121].

## 6. Conclusions

All subjects with AA should be referred to specialist centers to establish the most appropriate diagnosis, therapeutic procedure, and follow-up. A representative marrow biopsy is critical to the evaluation. In the initial work-up of all patients, chromosomal breakage testing, PNH flow cytometry, and telomere length by flow-FISH on a peripheral blood sample should be performed. However, the presence of PNH cells does not imply the PNH disease diagnosis since the clonal expansion of PNH is linked to AA. The identification of PNH clones should prompt a BM evaluation. The first-choice treatment of severe forms is HSCT if an identical HLA family donor is available, in pediatric patients, and in young adults up to 40 years and even beyond (50–60 years) if the subject is in good general condition and has no comorbidities. If an identical HLA family donor is not available, HSCT MUD is the first-choice treatment in patients up to 20 years of age if performed within 2–3 months of diagnosis and in subjects with severe disease, at high risk of infections, and with a young donor. In other cases, IST (equine ATG + CSA) is the treatment of first choice by virtue of the excellent chances of survival, even if it determines a lower quality of survival and carries a risk of late clonal evolution that is particularly high in young people and hence dictates lifelong surveillance. The addition of Eltrombopag improved the response of classical IST, although this has yet to be fully confirmed.

## Figures and Tables

**Table 1:** Exotoxins and compounds implicated in AA.

**Agents Implicated in AA**
Drugs *1.* *Antibiotics* CAF, Sulphonamide, cotrimoxazole, linezolid. *2.* *Antirheumatics* Gold salts Hydroxychloroquine Allopurinol Penicillamine. *3.* *Anticonvulsants* Phenytoin, carbamazepine. *4.* *Anti-inflammatory* Piroxicam, indomethacin, diclofenac, sulfasalazine, naproxen. *5.* *Antithyroid* Carbimazole, thiouracil.
**Chemicials** *1.* *Benzene* *2.* *Pesticides* Organochloride, organophosphates. *3.* *Narcotic drugs* Ecstasy, MDMA, methamphetamine.

**Table 2:** Acquired and inherited causes of bone marrow failure.

**Acquired**	**Inherited**
Acquired aplastic anemia *	Inherited bone marrow failure and myeloid malignancies predisposition syndromes (IBMF/MMPS) -Fanconi anemia -Short telomere syndromes -Shwachman-Diamond Syndrome -GATA2 deficiency -SAMD9/SAMD9L disorders
Hypocellular MDS	
Medications/toxins	
Anorexia nervosa	Inborn errors of immunity (i.e., X-linked lymphoproliferative disorder)

*Alternative underlying conditions inducing bone marrow failure should be excluded before approaching the patient with aplastic anemia.

## Data Availability

Not applicable.
